# Supplementary material for: Impact of Sapphire Step Height on the Growth of Monolayer Molybdenum Disulfide
Source: Nanomaterials (Basel). 2023 Nov 30;13(23):3056. doi: 10.3390/nano13233056 (PMC10707831; doi:10.3390/nano13233056)
Supplement: Supplementary file 1 [file nanomaterials-13-03056-s001.zip › nanomaterials-2709551-supplementary.pdf]

nanomaterials

MDPI

Supplementary Materials

Impact of Sapphire Step Height on the Growth of Monolayer Molybdenum Disulfide

Jie Lu[1,†], Miaomiao Zheng[1,†], Jinxin Liu[1], Yufeng Zhang[1], Xueao Zhang[1,2,*] and Weiwei Cai[1,2,*]

1 College of Physical Science and Technology, Xiamen University, Xiamen 361005, China;
2 Jiujiang research institute of Xiamen University, Jiujiang 360404, China
* Correspondence: xazhang@xmu.edu.cn (X.Z.); wwcai@xmu.edu.cn (W.C.).
† These authors contributed equally to this work.

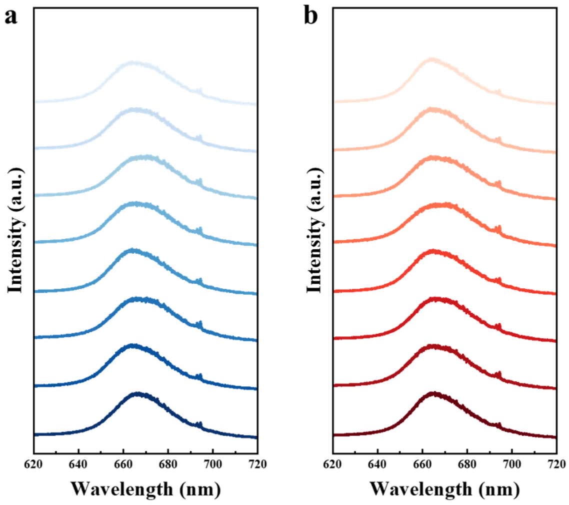

Figure S1. PL spectra of MoS_2 with triangle (a) and nanoribbon (b).

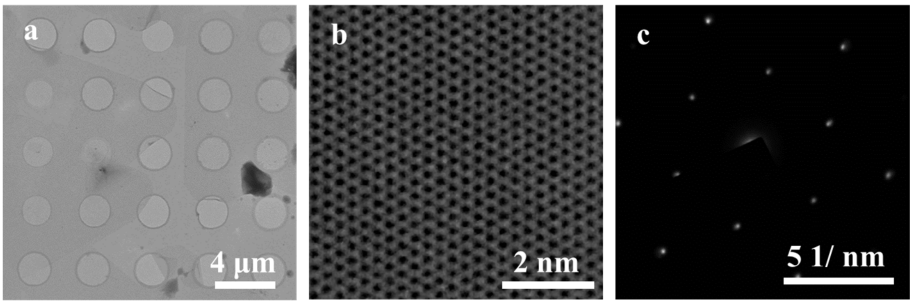

Figure S2. The crystallinity of triangle MoS_2. (a) Low-magnification TEM image corresponding high-resolution TEM image (b) and SAED pattern (c).

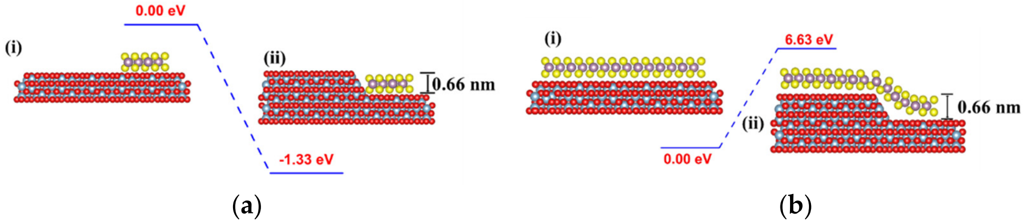

Figure S3. CP2K calculations about the growth. (a) Energy profiles of MoS_2 on a flat surface (i), attached to a 0.66 nm-high step (ii). (b) Energy profiles of MoS_2 on a flat surface (i), crossing a 0.66 nm-high step (ii). The binding energies of MoS_2 on a flat surface are set as energy references.
